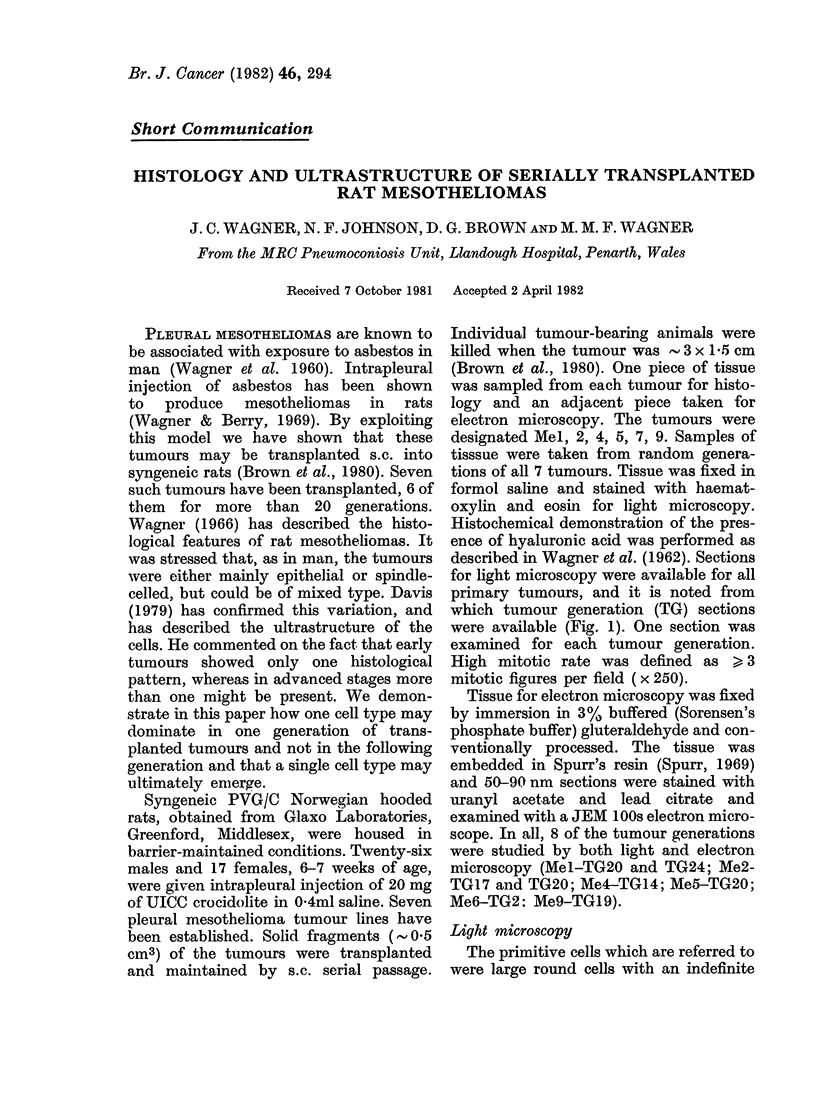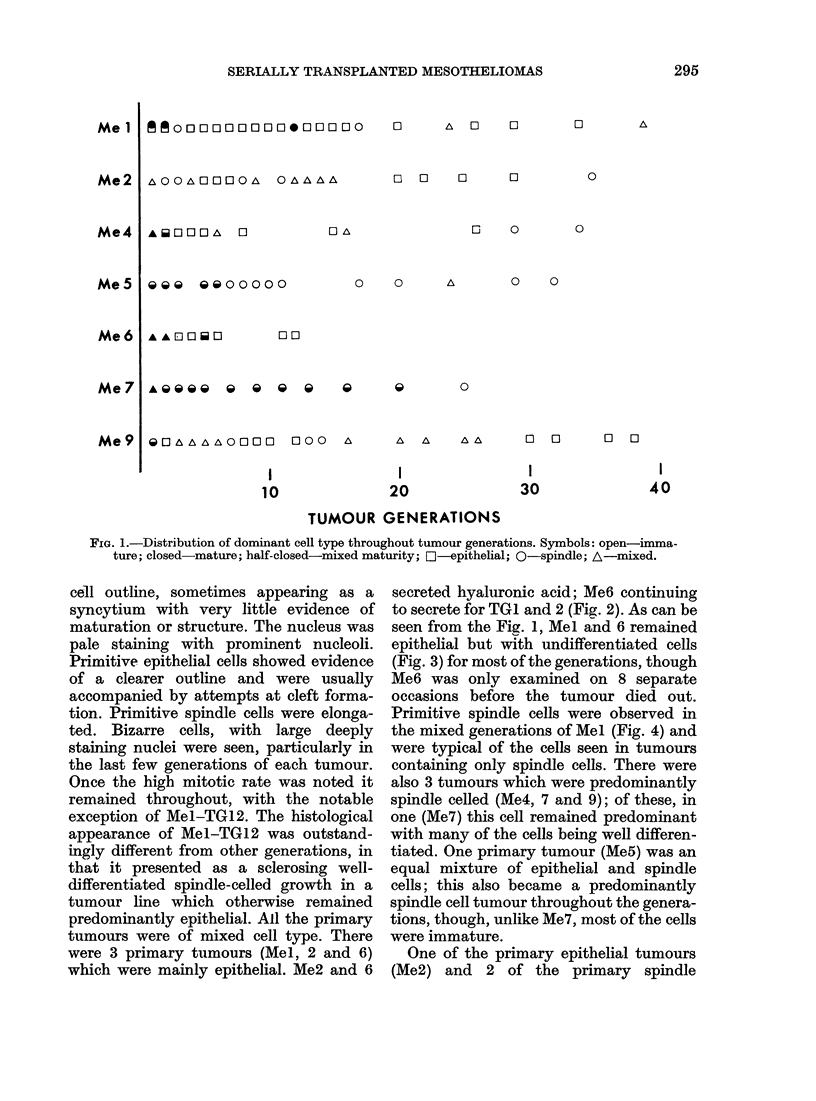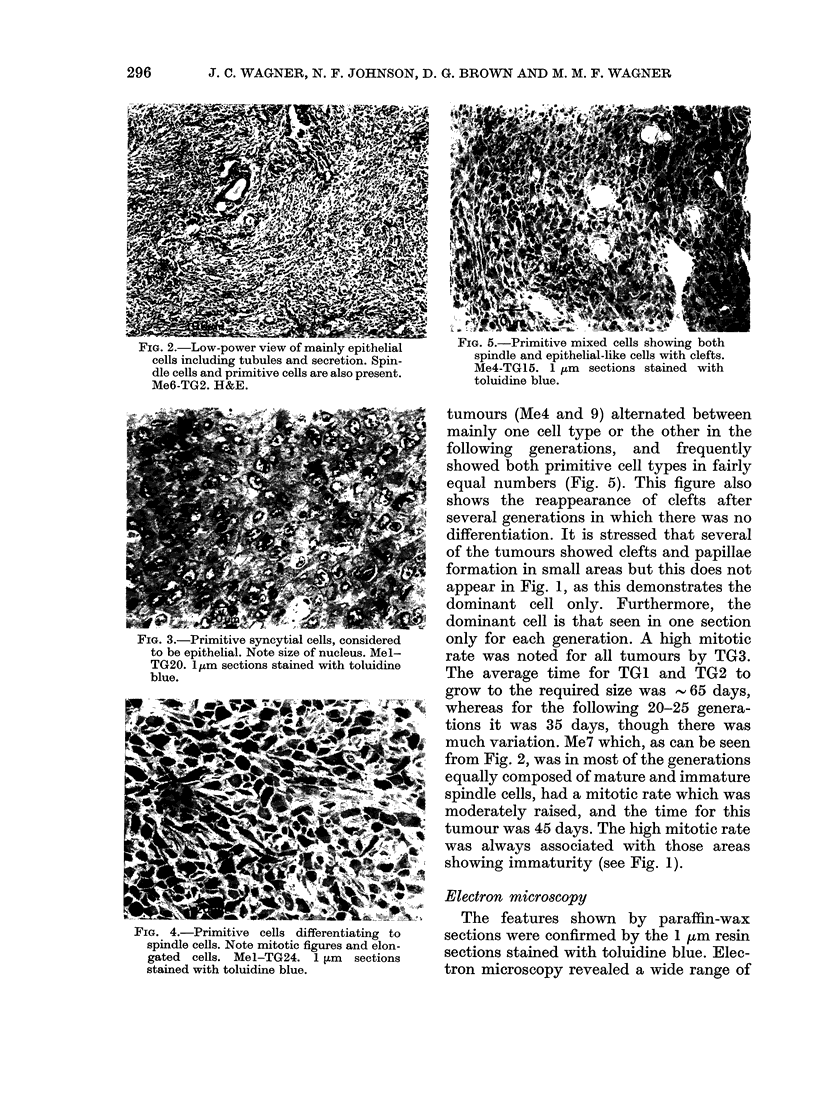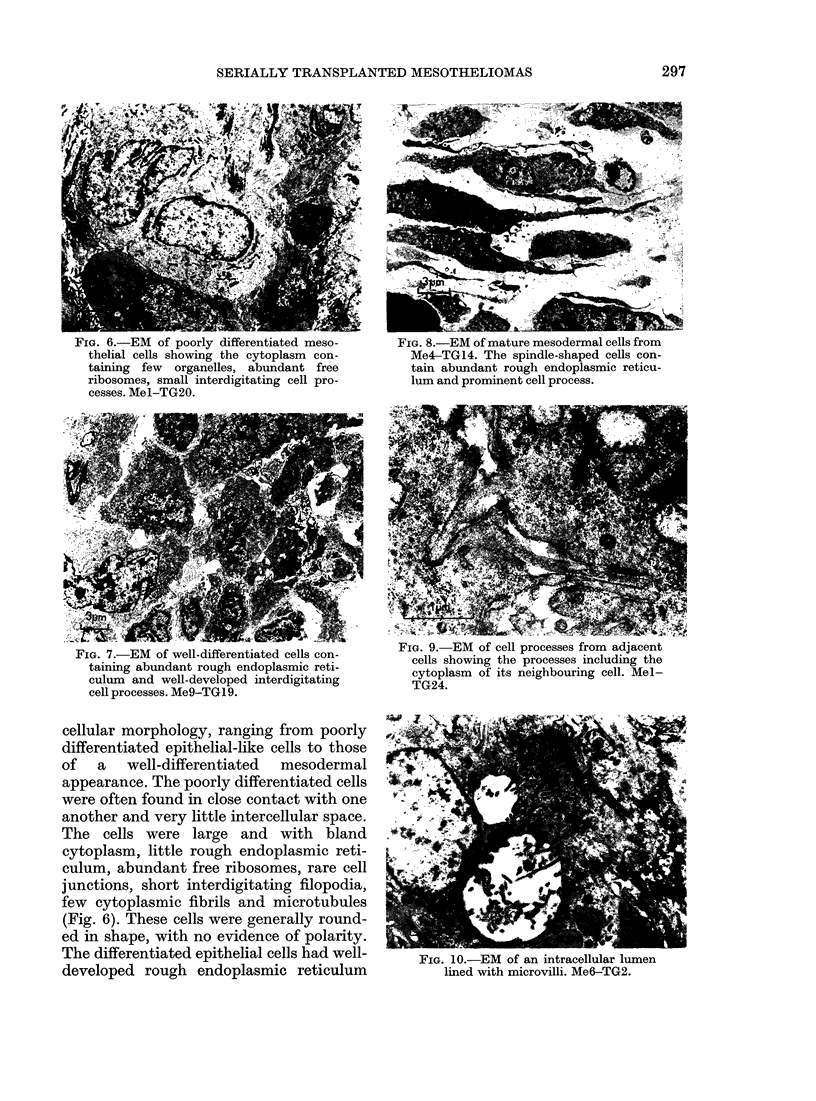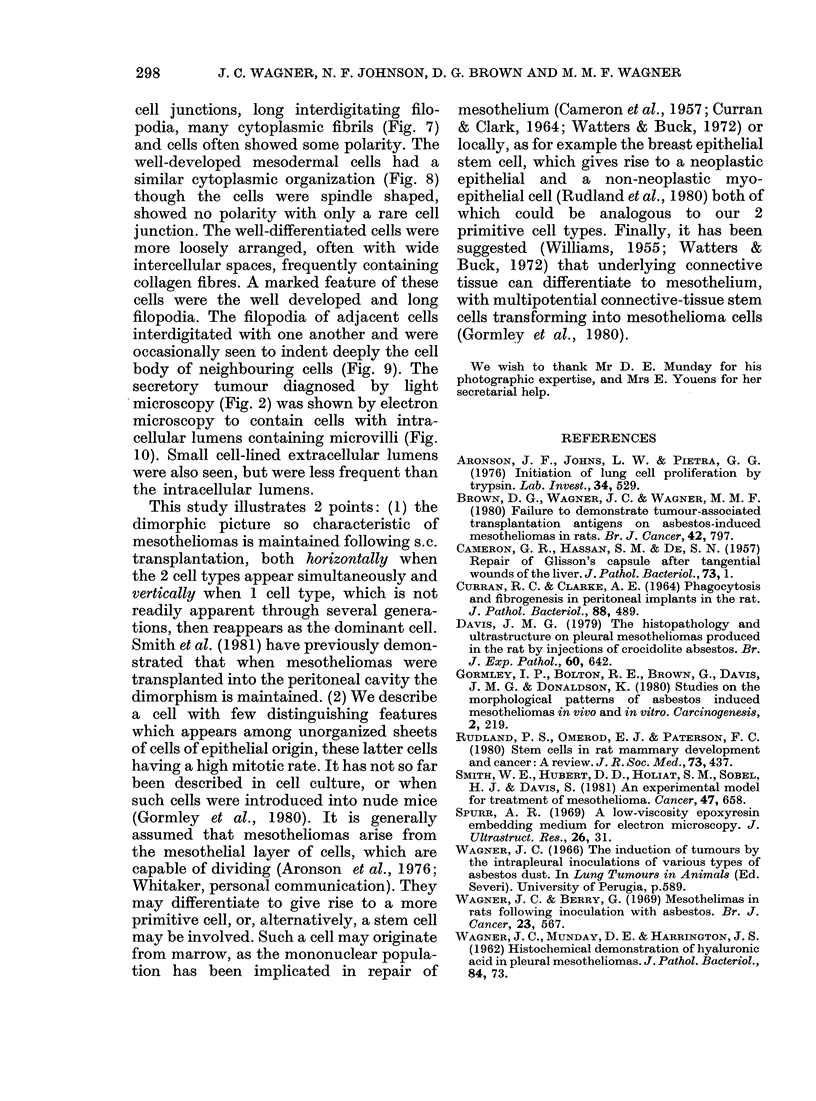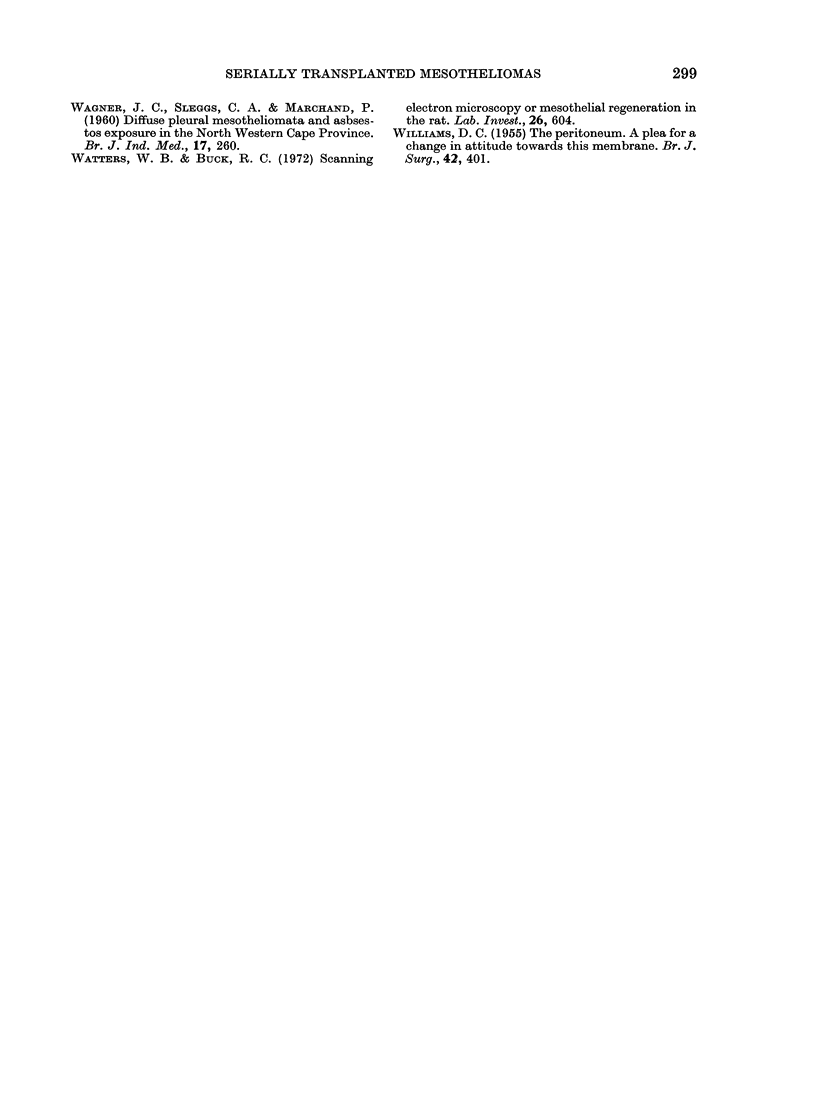# Histology and ultrastructure of serially transplanted rat mesotheliomas.

**DOI:** 10.1038/bjc.1982.197

**Published:** 1982-08

**Authors:** J. C. Wagner, N. F. Johnson, D. G. Brown, M. M. Wagner

## Abstract

**Images:**


*Br. J. Cancer* (1982) **46**, 294

***Short Communication***

### HISTOLOGY AND ULTRASTRUCTURE OF SERIALLY TRANSPLANTED RAT MESOTHELIOMAS

J. C. WAGNER, N. F. JOHNSON, D. G. BROWN AND M. M. F. WAGNER

*From the MRC Pneumoconiosis Unit, Llandough Hospital, Penarth, Wales*

Received 7 October 1981 Accepted 2 April 1982

PLEURAL MESOTHELIOMAS are known to be associated with exposure to asbestos in man (Wagner *et al.* 1960). Intrapleural injection of asbestos has been shown to produce mesotheliomas in rats (Wagner & Berry, 1969). By exploiting this model we have shown that these tumours may be transplanted s.c. into syngeneic rats (Brown *et al.*, 1980). Seven such tumours have been transplanted, 6 of them for more than 20 generations. Wagner (1966) has described the histological features of rat mesotheliomas. It was stressed that, as in man, the tumours were either mainly epithelial or spindle-celled, but could be of mixed type. Davis (1979) has confirmed this variation, and has described the ultrastructure of the cells. He commented on the fact that early tumours showed only one histological pattern, whereas in advanced stages more than one might be present. We demonstrate in this paper how one cell type may dominate in one generation of transplanted tumours and not in the following generation and that a single cell type may ultimately emerge.

Syngeneic PVG/C Norwegian hooded rats, obtained from Glaxo Laboratories, Greenford, Middlesex, were housed in barrier-maintained conditions. Twenty-six males and 17 females, 6–7 weeks of age, were given intrapleural injection of 20 mg of UICC crocidolite in 0·4ml saline. Seven pleural mesothelioma tumour lines have been established. Solid fragments ($\sim$0·5 cm$^3$) of the tumours were transplanted and maintained by s.c. serial passage. Individual tumour-bearing animals were killed when the tumour was $\sim 3 \times 1{\cdot}5$ cm (Brown *et al.*, 1980). One piece of tissue was sampled from each tumour for histology and an adjacent piece taken for electron microscopy. The tumours were designated Mel, 2, 4, 5, 7, 9. Samples of tisssue were taken from random generations of all 7 tumours. Tissue was fixed in formol saline and stained with haematoxylin and eosin for light microscopy. Histochemical demonstration of the presence of hyaluronic acid was performed as described in Wagner *et al.* (1962). Sections for light microscopy were available for all primary tumours, and it is noted from which tumour generation (TG) sections were available (Fig. 1). One section was examined for each tumour generation. High mitotic rate was defined as $\geqslant 3$ mitotic figures per field ($\times 250$).

Tissue for electron microscopy was fixed by immersion in 3% buffered (Sorensen's phosphate buffer) gluteraldehyde and conventionally processed. The tissue was embedded in Spurr's resin (Spurr, 1969) and 50–90 nm sections were stained with uranyl acetate and lead citrate and examined with a JEM 100s electron microscope. In all, 8 of the tumour generations were studied by both light and electron microscopy (Mel–TG20 and TG24; Me2-TG17 and TG20; Me4–TG14; Me5–TG20; Me6–TG2: Me9–TG19).

### *Light microscopy*

The primitive cells which are referred to were large round cells with an indefinite

FIG. 1.—Distribution of dominant cell type throughout tumour generations. Symbols: open—immature; closed—mature; half-closed—mixed maturity; □—epithelial; ○—spindle; △—mixed.

cell outline, sometimes appearing as a syncytium with very little evidence of maturation or structure. The nucleus was pale staining with prominent nucleoli. Primitive epithelial cells showed evidence of a clearer outline and were usually accompanied by attempts at cleft formation. Primitive spindle cells were elongated. Bizarre cells, with large deeply staining nuclei were seen, particularly in the last few generations of each tumour. Once the high mitotic rate was noted it remained throughout, with the notable exception of Me1–TG12. The histological appearance of Me1–TG12 was outstandingly different from other generations, in that it presented as a sclerosing well-differentiated spindle-celled growth in a tumour line which otherwise remained predominantly epithelial. All the primary tumours were of mixed cell type. There were 3 primary tumours (Me1, 2 and 6) which were mainly epithelial. Me2 and 6 secreted hyaluronic acid; Me6 continuing to secrete for TG1 and 2 (Fig. 2). As can be seen from the Fig. 1, Me1 and 6 remained epithelial but with undifferentiated cells (Fig. 3) for most of the generations, though Me6 was only examined on 8 separate occasions before the tumour died out. Primitive spindle cells were observed in the mixed generations of Me1 (Fig. 4) and were typical of the cells seen in tumours containing only spindle cells. There were also 3 tumours which were predominantly spindle celled (Me4, 7 and 9); of these, in one (Me7) this cell remained predominant with many of the cells being well differentiated. One primary tumour (Me5) was an equal mixture of epithelial and spindle cells; this also became a predominantly spindle cell tumour throughout the generations, though, unlike Me7, most of the cells were immature.

One of the primary epithelial tumours (Me2) and 2 of the primary spindle

FIG. 2.—Low-power view of mainly epithelial cells including tubules and secretion. Spindle cells and primitive cells are also present. Me6-TG2. H&E.

FIG. 3.—Primitive syncytial cells, considered to be epithelial. Note size of nucleus. Me1–TG20. 1 µm sections stained with toluidine blue.

FIG. 4.—Primitive cells differentiating to spindle cells. Note mitotic figures and elongated cells. Me1–TG24. 1 µm sections stained with toluidine blue.

FIG. 5.—Primitive mixed cells showing both spindle and epithelial-like cells with clefts. Me4-TG15. 1 µm sections stained with toluidine blue.

tumours (Me4 and 9) alternated between mainly one cell type or the other in the following generations, and frequently showed both primitive cell types in fairly equal numbers (Fig. 5). This figure also shows the reappearance of clefts after several generations in which there was no differentiation. It is stressed that several of the tumours showed clefts and papillae formation in small areas but this does not appear in Fig. 1, as this demonstrates the dominant cell only. Furthermore, the dominant cell is that seen in one section only for each generation. A high mitotic rate was noted for all tumours by TG3. The average time for TG1 and TG2 to grow to the required size was ~65 days, whereas for the following 20–25 generations it was 35 days, though there was much variation. Me7 which, as can be seen from Fig. 2, was in most of the generations equally composed of mature and immature spindle cells, had a mitotic rate which was moderately raised, and the time for this tumour was 45 days. The high mitotic rate was always associated with those areas showing immaturity (see Fig. 1).

*Electron microscopy*

The features shown by paraffin-wax sections were confirmed by the 1 µm resin sections stained with toluidine blue. Electron microscopy revealed a wide range of

FIG. 6.—EM of poorly differentiated mesothelial cells showing the cytoplasm containing few organelles, abundant free ribosomes, small interdigitating cell processes. Me1–TG20.

FIG. 7.—EM of well-differentiated cells containing abundant rough endoplasmic reticulum and well-developed interdigitating cell processes. Me9–TG19.

cellular morphology, ranging from poorly differentiated epithelial-like cells to those of a well-differentiated mesodermal appearance. The poorly differentiated cells were often found in close contact with one another and very little intercellular space. The cells were large and with bland cytoplasm, little rough endoplasmic reticulum, abundant free ribosomes, rare cell junctions, short interdigitating filopodia, few cytoplasmic fibrils and microtubules (Fig. 6). These cells were generally rounded in shape, with no evidence of polarity. The differentiated epithelial cells had well-developed rough endoplasmic reticulum

FIG. 8.—EM of mature mesodermal cells from Me4–TG14. The spindle-shaped cells contain abundant rough endoplasmic reticulum and prominent cell process.

FIG. 9.—EM of cell processes from adjacent cells showing the processes including the cytoplasm of its neighbouring cell. Me1–TG24.

FIG. 10.—EM of an intracellular lumen lined with microvilli. Me6–TG2.

cell junctions, long interdigitating filopodia, many cytoplasmic fibrils (Fig. 7) and cells often showed some polarity. The well-developed mesodermal cells had a similar cytoplasmic organization (Fig. 8) though the cells were spindle shaped, showed no polarity with only a rare cell junction. The well-differentiated cells were more loosely arranged, often with wide intercellular spaces, frequently containing collagen fibres. A marked feature of these cells were the well developed and long filopodia. The filopodia of adjacent cells interdigitated with one another and were occasionally seen to indent deeply the cell body of neighbouring cells (Fig. 9). The secretory tumour diagnosed by light microscopy (Fig. 2) was shown by electron microscopy to contain cells with intracellular lumens containing microvilli (Fig. 10). Small cell-lined extracellular lumens were also seen, but were less frequent than the intracellular lumens.

This study illustrates 2 points: (1) the dimorphic picture so characteristic of mesotheliomas is maintained following s.c. transplantation, both *horizontally* when the 2 cell types appear simultaneously and *vertically* when 1 cell type, which is not readily apparent through several generations, then reappears as the dominant cell. Smith *et al.* (1981) have previously demonstrated that when mesotheliomas were transplanted into the peritoneal cavity the dimorphism is maintained. (2) We describe a cell with few distinguishing features which appears among unorganized sheets of cells of epithelial origin, these latter cells having a high mitotic rate. It has not so far been described in cell culture, or when such cells were introduced into nude mice (Gormley *et al.*, 1980). It is generally assumed that mesotheliomas arise from the mesothelial layer of cells, which are capable of dividing (Aronson *et al.*, 1976; Whitaker, personal communication). They may differentiate to give rise to a more primitive cell, or, alternatively, a stem cell may be involved. Such a cell may originate from marrow, as the mononuclear population has been implicated in repair of mesothelium (Cameron *et al.*, 1957; Curran & Clark, 1964; Watters & Buck, 1972) or locally, as for example the breast epithelial stem cell, which gives rise to a neoplastic epithelial and a non-neoplastic myoepithelial cell (Rudland *et al.*, 1980) both of which could be analogous to our 2 primitive cell types. Finally, it has been suggested (Williams, 1955; Watters & Buck, 1972) that underlying connective tissue can differentiate to mesothelium, with multipotential connective-tissue stem cells transforming into mesothelioma cells (Gormley *et al.*, 1980).

We wish to thank Mr D. E. Munday for his photographic expertise, and Mrs E. Youens for her secretarial help.

### REFERENCES

Aronson, J. F., Johns, L. W. & Pietra, G. G. (1976) Initiation of lung cell proliferation by trypsin. *Lab. Invest.*, **34**, 529.

Brown, D. G., Wagner, J. C. & Wagner, M. M. F. (1980) Failure to demonstrate tumour-associated transplantation antigens on asbestos-induced mesotheliomas in rats. *Br. J. Cancer*, **42**, 797.

Cameron, G. R., Hassan, S. M. & De, S. N. (1957) Repair of Glisson's capsule after tangential wounds of the liver. *J. Pathol. Bacteriol.*, **73**, 1.

Curran, R. C. & Clarke, A. E. (1964) Phagocytosis and fibrogenesis in peritoneal implants in the rat. *J. Pathol. Bacteriol.*, **88**, 489.

Davis, J. M. G. (1979) The histopathology and ultrastructure on pleural mesotheliomas produced in the rat by injections of crocidolite absestos. *Br. J. Exp. Pathol.*, **60**, 642.

Gormley, I. P., Bolton, R. E., Brown, G., Davis, J. M. G. & Donaldson, K. (1980) Studies on the morphological patterns of asbestos induced mesotheliomas *in vivo* and *in vitro*. *Carcinogenesis*, **2**, 219.

Rudland, P. S., Omerod, E. J. & Paterson, F. C. (1980) Stem cells in rat mammary development and cancer: A review. *J. R. Soc. Med.*, **73**, 437.

Smith, W. E., Hubert, D. D., Holiat, S. M., Sobel, H. J. & Davis, S. (1981) An experimental model for treatment of mesothelioma. *Cancer*, **47**, 658.

Spurr, A. R. (1969) A low-viscosity epoxyresin embedding medium for electron microscopy. *J. Ultrastruct. Res.*, **26**, 31.

Wagner, J. C. (1966) The induction of tumours by the intrapleural inoculations of various types of asbestos dust. In *Lung Tumours in Animals* (Ed. Severi). University of Perugia, p.589.

Wagner, J. C. & Berry, G. (1969) Mesothelimas in rats following inoculation with asbestos. *Br. J. Cancer*, **23**, 567.

Wagner, J. C., Munday, D. E. & Harrington, J. S. (1962) Histochemical demonstration of hyaluronic acid in pleural mesotheliomas. *J. Pathol. Bacteriol.*, **84**, 73.

WAGNER, J. C., SLEGGS, C. A. & MARCHAND, P. (1960) Diffuse pleural mesotheliomata and asbsestos exposure in the North Western Cape Province. *Br. J. Ind. Med.*, **17**, 260.
WATTERS, W. B. & BUCK, R. C. (1972) Scanning electron microscopy or mesothelial regeneration in the rat. *Lab. Invest.*, **26**, 604.
WILLIAMS, D. C. (1955) The peritoneum. A plea for a change in attitude towards this membrane. *Br. J. Surg.*, **42**, 401.